# Applicability of *Hibiscus sabdariffa* L. Extract With Anti‐Inflammatory Potential in Human Health: A Review

**DOI:** 10.1002/fsn3.70938

**Published:** 2025-09-23

**Authors:** Anne Caroline Silva Nogueira da Cruz, Michelline Joana Tenório Albuquerque Madruga Mesquita, Guilherme kauan Rocha Dantas, Eduardo Rodrigues Silva, Victória Carvalho Falcone De Oliveira, Heitor Martins Rezende, Gustavo Medeiros Frota, Mariana Cavalcante, José Renzo Castro Garcês, Marcelo Souza de Andrade, Maria do Socorro de Sousa Cartagenes

**Affiliations:** ^1^ Postgraduate Program in Health Sciences Federal University of Maranhão São Luís Maranhão Brazil; ^2^ Department of Medicine Federal University of Maranhão São Luís Maranhão Brazil; ^3^ Postgraduate Program in Biotechnology Federal University of Maranhão São Luís Maranhão Brazil; ^4^ Postgraduate Program in Adult Health Federal University of Maranhão São Luís Maranhão Brazil

**Keywords:** anti‐inflammatory, bioactive compounds, *Hibiscus sabdariffa* L, human health

## Abstract

Studies demonstrate the health benefits of *H. sabdariffa* L., highlighting its anti‐inflammatory activity. Therefore, it is essential to expand studies on this plant and its various applications, aiming to maximize its benefits for human health, especially through the use of the bioactive compounds present in *Hibiscus sabdariffa* L. To prepare this review article, we conducted a literature search through virtual libraries of scientific articles, such as LILACS, PUBMED, and BVS, using the following descriptors: *Hibiscus sabdariffa* L. and anti‐inflammatory. The selected publications cover the period 2019 to 2024 (5 years). A total of 25 articles were found, distributed as follows: PUBMED: 24 articles, of which 11 were excluded. The exclusion was because these articles did not meet the research objective or were review articles that did not meet the established criteria. No articles were found in the LILACS database, indicating that the search did not return relevant results within the search criteria. One article was found in the VHL database, but it was excluded due to duplication with an article already found in the PUBMED database. Analysis of the studies selected in the scientific database highlights the benefits of using *Hibiscus sabdariffa* L. in the treatment of various pathologies, such as degenerative diseases, intestinal dysfunctions, and atherosclerosis, among others, with a particular emphasis on its anti‐inflammatory activities. However, the results did not demonstrate benefits in a study focused on wound healing, indicating that further studies are needed to investigate its specific application.

## Graphical abstract

O artigo de revisão explora os potenciais benefícios anti‐inflamatórios do extrato de *Hibiscus sabdariffa* L. para a saúde humana. O objetivo é fornecer uma análise detalhada das propriedades terapêuticas da planta e sua aplicabilidade no tratamento de condições relacionadas à inflamação.

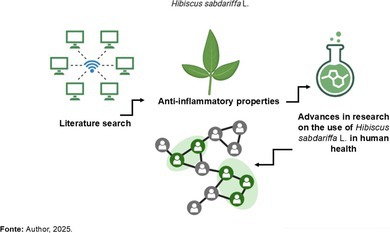

## 1 Introduction

In recent years, the focus on plant research has increased globally to uncover the immense potential of medicinal plants used in various traditional systems. Several medicinal plants have been studied and can be used as potent phytochemical agents in the therapeutic treatment of various diseases; one of them is *Hibiscus sabdariffa* L. (HS) – known for its delicacy and medicinal properties that bring several health benefits (Riaz and Chopra 2018) Several studies have shown that *H. sabdariffa calyxes* contain bioactive compounds (BCs) responsible for the therapeutic effects in such extracts, such as organic acids, anthocyanins, flavonoids, and phenolic acids (Ojulari et al. 2019; Sáyago‐Ayerdi et al. 2021).

Red rosewood ( *Hibiscus sabdariffa* L., Malvaceae) is native to Africa and widely cultivated in tropical climates. Hibiscus calyxes are recognized for their nutraceutical properties and multiple health‐promoting effects, including reproductive health (Okafor et al. 2020), antioxidant, anti‐inflammatory, and antihyperlipidemic activities (Laskar and Mazumder 2020).

Recent studies have also proposed *H. sabdariffa* as a neuroprotective agent to prevent Alzheimer's disease in mice (El‐Shiekh et al. 2020). The pharmacological effects of this plant have been linked to its anthocyanins and its estrogenic properties (Morales‐Luna et al. 2019).

HS leaves are a good source of various nutrients like protein, fat, carbohydrates, phosphorus, iron, β‐carotene, riboflavin, and ascorbic acid. They contain high levels of polyphenolic compounds, mainly chlorogenic acid and its isomers quercetin and kaempferol glycosides that contribute to antioxidant capacity and anti‐inflammatory activity (Riaz and Chopra 2018). They contain major subclasses of flavonoids, such as rutin, quercetin, and kaempferol, as well as their derivatives (Gosain et al. 2010; Lyu et al. 2020). In addition, they have also been identified as containing neochlorogenic acid, chlorogenic acid, cryptochlorogenic acid (Wang et al. 2014), as well as protocatechuic acid and sitosterol‐β‐D‐galactoside (Gosain et al. 2010).

They also have a higher concentration of polyphenolic compounds, especially chlorogenic acid, quercetin, and kaempferol, contributing to antioxidant capacity and anti‐inflammatory activity (Zhen et al. 2016). They also exhibit antimicrobial and potent free radical scavenger activity against reactive oxygen species (ROS) (Ochani and Mello 2009). Anthocyanins have also been investigated to have anti‐inflammatory potential by reducing levels of inflammatory mediators (Shen et al. 2016).

The mechanisms involved in the anti‐inflammatory activities of *H. sabdariffa* extracts appear to be multifunctional, involving different bioactive agents that can interact with various biological targets to elicit the observed anti‐inflammatory effects. Recent studies have demonstrated the beneficial effects of aqueous extracts of hibiscus (Agunbiade et al. 2022; Singh et al. 2022). Hydroxycitric acid and hibiscus acid are the most abundant organic acids, while chlorogenic acid is the main phenolic acid detected in aqueous extracts of *H. sabdariffa* .

*Hibiscus sabdariffa* has anti‐inflammatory effects attributed to the presence of polyphenolic compounds with significant pharmacological properties. Flavonoids present in the plant also demonstrate the ability to inhibit nuclear transcription factor kappa B (NF‐κB), essential in mediating inflammatory responses (Ekka and Ahirwar 2025).

Some studies show the health benefits of *H. sabdariffa* , such as its anti‐inflammatory activity (Olaokun and Mkolo 2020; Umeoguaju et al. 2021). The phytochemical characterization of a drink of *H. sabdariffa* prepared by decoction with the calyxes of hibiscus and citric acid, mint (added at the beginning of the preparation of the drink from dried and crushed mint leaves) and stevia as a sweetener revealed the richness of this infusion with 35 bioactive compounds, compared to 11 of a product already available on the market. Hibiscus acid was the main organic acid, along with hydroxycitric acid (Sáyago‐Ayerdi et al. 2021; Rodríguez‐Romero et al. 2023).

Several studies have explored the use of extracts of *Hibiscus sabdariffa* L. and its subspecies, investigating different forms of administration of the plant for human benefit, being in studies with aqueous and hydroalcoholic extracts, with leaves, calyxes, or whole plant. In this context, the objective of this review is to gather research on the relationship of *Hibiscus sabdariffa* L. and human health, with emphasis on its anti‐inflammatory effect and highlighting the variety and forms of use of the plant and its possible therapeutic applications, in the period from 2019 to 2024.

## 2 Methods

This is a qualitative study, with specific scientific productions on the study with *Hibiscus sabdariffa* L. extract and its contribution to human health, with an emphasis on its anti‐inflammatory properties. The literature search was carried out through virtual libraries of scientific articles, such as LILACS, PUBMED, and BVS, with the following descriptors: *Hibiscus sabdariffa* L. and anti‐inflammatory, with publications limited to the period from 2019 to 2024 (5 years), reflecting the most recent advances in the area of research on the use of *Hibiscus*.

**FIGURE 1:**
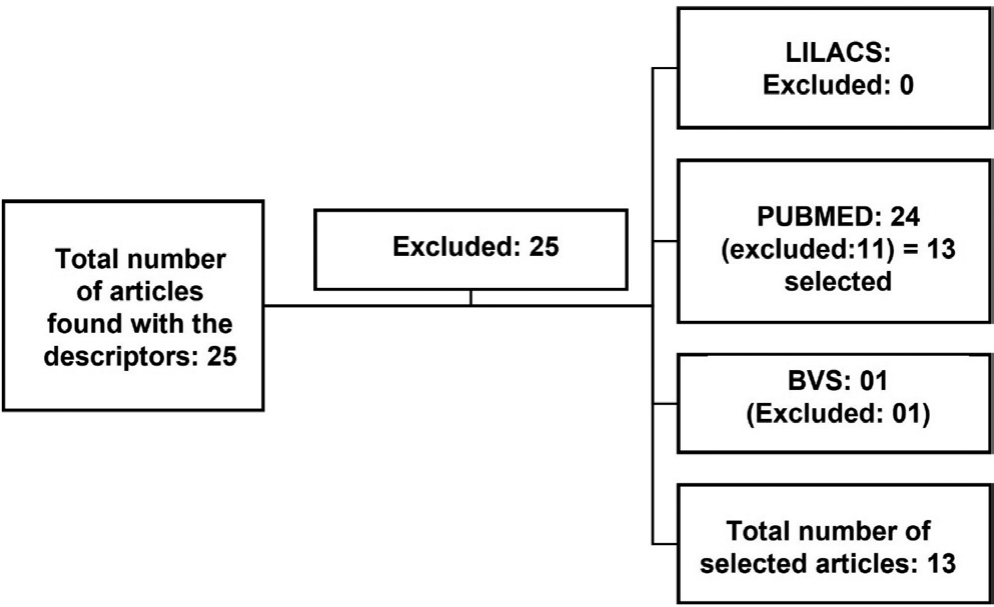
Flowchart of the method of selection of articles for the literature review from 2019 to 2024. *Source:* Author 2025.

In the search performed for the combination of descriptor operators, the AND operator was used, as described (Figure 1). Review articles, duplicate articles in another database, articles that did not answer the question or objective of the review, articles that dealt with other plant subspecies, and articles on soil/phylogeny/composition were excluded from this review.

According to the inclusion and exclusion criteria defined for the search in the main scientific databases used by the academic community, a survey was carried out in the three main databases, where a total of 25 articles were found, distributed as follows: PUBMED: 24 articles were found, of which 11 were excluded. The reason for the exclusion was that these articles did not meet the objective of the research or were review articles, which did not fit the established criteria. No article was found in the LILACS database, indicating that the search did not return relevant results within the search criteria. In the VHL, 1 article was found; however, it was excluded due to duplicity with an article already found in the PUBMED database.

## 3 Results

In this section, the main results found in the analyzed publications are presented, focusing on the relationship between *Hibiscus sabdariffa* L. and human health, especially with regard to anti‐inflammatory properties, in the period from 2019 to 2024, as described below in Table 1.

**TABLE 1:** Methodological description of the articles from 2019 to 2024 included in this literature review.

Author (year)	Title	Conclusion
Idowu‐Adebayo et al. (2021)	Enrichment of the drink zobo ( *Hibiscus sabdariffa* ) sold on the streets with turmeric (Turmeric longa) to increase its beneficial health properties	Turmeric‐fortified zobo may play a role in a healthy diet for its health‐supporting properties. Consumption of a typical 500 mL serving (representative bottle size of zobo beverage packaged by street vendors in Nigeria) of turmeric‐fortified zobo would contribute 63%–88% DV and 18%–23% DV of iron and zinc. Overall, fortification with boiled turmeric improves the antioxidant and nutritional quality of zobo, specifically in relation to vitamin C, delphinidin‐3‐sambubioside, and iron.
Chiaino et al. (2020)	Preparation based on extracts of olive leaves and hibiscus flowers protects the brain from oxidative stress‐induced injury	The present results suggest the possibility of PRES (*Pres phytum* (PRES) is a nutraceutical product composed of leaf and flower extracts of *Olea europaea* L. and *Hibiscus sabdariffa* L.) as a nutraceutical, which may help in the prevention of neurodegenerative diseases.
Alsharif et al. (2021)	Protocatechuic acid attenuates lipopolysaccharide‐induced septic lung injury in mice: the possible role through the suppression of oxidative stress, inflammation, and apoptosis	The results recorded showed that the pre‐administration of PCA (protocatechuic acid) was able to significantly nullify the damage to the septic response associated with lung tissue. This protective effect comes from its strong antioxidant, anti‐inflammatory, and anti‐apoptotic activities, suggesting that PCA can be applied to alleviate ALI associated with the development of sepsis.
Janson et al. (2021)	The calyx extract of *Hibiscus sabdariffa* L. prevents adipogenesis of 3 T3‐L1 adipocytes and obesity‐related insulin resistance in obese rats induced by high‐fat diet.	Rosella can prevent lipid accumulation by suppressing 3 T3‐L1 adipocyte differentiation by downregulating the expression of the adipogenic gene. The results of this study demonstrated that the molecular mechanism underlying the protective effect of rosella may be an alternative approach to the prevention of obesity‐related insulin resistance.
Wang et al. (2022)	Potential of *Hibiscus sabdariffa* L. and hibiscus acid to reverse skin aging.	Assays have indicated that hibiscus acid treatment can reduce extracellular ATP secretion and carbonyl protein production, as well as maintain a high level of reduced/oxidized glutathione in skin cells, thus providing a possible mechanism by which hibiscus acid can combat antioxidant stress.
Bernardes et al. (2023)	Hibiscus ( *Hibiscus sabdariffa* L.) increases butyrate synthesis and reduces inflammatory cells, attenuating the formation of aberrant crypt foci in BALB/c mice induced to preneoplastic lesions.	Supplementation of dehydrated calyxes of *H. sabdariffa* DHSC may be recommended to attenuate cellular responses in the early stage of preneoplastic lesions.
Mattioli et al. (2022)	Olea europea L. *leaf extracts* and *Hibiscus sabdariffa* L. *petals*: cardiovascular network herbal blend, target of intestinal motility dysfunction application	HM can control ileum and colon contractility without blocking bolus progression, can selectively inhibit iNOS, and has strong pro‐apoptotic activity against Caco‐2 cells.
Rambe et al. (2022)	The effect of rosella leaf extract gel ( *Hibiscus sabdariffa* L.) in wound healing.	There were no significant differences in wound healing and epithelial thickness between the groups. Rosella leaf extract at a concentration of 15% showed higher healing properties based on clinical and histological evaluation. Although there were no statistically significant differences, the rosella leaf showed an opportunity to be investigated as a potential wound healing therapy.
Malacrida et al. (2022)	Evaluation of the antitumor effect of *Hibiscus sabdariffa* extract on human breast cancer cells.	HsEF ( *Hibiscus sabdariffa* Extract Fraction) has antitumor effects on both breast tumor cells examined, and the involvement of ERα could explain the differences observed between the two cell lines.
Sun et al. 2022	Delphinidin‐3‐Glucoside, an active compound in the calyxes of *Hibiscus sabdariffa* , inhibits oxidative stress and inflammation in rabbits with atherosclerosis.	DP (Delphinidin‐3‐glucoside) relieved oxidative stress and inflammation induced by DH (high‐fat diet) in rabbits with atherosclerosis. These results provided the scientific basis for the development of new therapies.
El‐Shiekh et al. (2020)	*Hibiscus sabdariffa* L.: A potent natural neuroprotective agent for the prevention of streptozotocin‐induced Alzheimer's disease in mice.	Hibiscus prevented memory impairment, and this can be attributed to STZ‐induced improvement in neuroinflammation and amyloidogenesis. Consequently, hibiscus represents a safe and promising agent that can be repurposed for AD (Alzheimer's disease) through the exercise of anti‐inflammatory, anti‐acetylcholinesterase, antioxidant, and anti‐amyloidogenic activities.
Arce‐Reynoso et al. (2023)	Bioavailability of bioactive compounds in *Hibiscus sabdariffa* beverage as anti‐inflammatory potential.	The microbiota extensively biotransformed the PCs (phenolic compounds), and their quantity was lower than that of organic acids, particularly hibiscus acid and hydroxycitric acid. Colonic metabolites derived from PCs and organic acids would be behind the anti‐inflammatory bioactivity described for *Hibiscus sabdariffa* L.
Huang et al. (2023)	Anticancer effects of gossypetin from *Hibiscus sabdariffa* in oral squamous cell carcinoma.	The results showed that gossypetin inhibits the proliferation, migration, and invasion of CECO cells and triggers apoptosis and cell cycle arrest in the CECO. Therefore, gossypetin has potential for use as a chemopreventive agent in oral cancer.

*Source:* Author 2024.

In general, the analysis of the studies selected in the scientific database shows the benefits of the use of *Hibiscus sabdariffa* L. in the treatment of various pathologies, with a focus on anti‐inflammatory activities. Chiaino et al. (2020) analyzed the effects of an olive leaf extract and *Hibiscus‐based* preparation on protecting the brain against oxidative stress‐induced injury, demonstrating that the combination of these extracts has a significant neuroprotective effect, protecting brain cells against oxidative stress.

The relationship between the use of *Hibiscus sabdariffa* L. and obesity treatments is already well established. When reviewing the database for this study, we identified a study carried out by Janson et al. (2021), which investigated whether the calyx extract of *Hibiscus sabdariffa* L. could prevent adipogenesis of 3 T3‐L1 adipocytes and obesity‐related insulin resistance in obese rats induced by a high‐fat diet. The results indicated that *Hibiscus* has the potential to prevent lipid accumulation, acting effectively in the suppression of 3 T3‐L1 adipocyte differentiation. This effect occurs through the downregulation of the expression of adipogenic genes, which are responsible for the formation and development of fat cells.

A study conducted by Alsharif et al. (2021), which investigated the effects of protocatechuic acid on LPS‐induced septic lung injury in mice, demonstrated that this acid is able to reduce the levels of reactive oxygen species, which are responsible for causing oxidative stress and cell damage. In addition, it also modulated the inflammatory response, reducing the expression of proinflammatory cytokines and the infiltration of inflammatory cells into the lungs of mice. The study suggests that the acid has therapeutic potential in protecting lung lesions caused by sepsis by reducing oxidative stress.

Hibiscus *supplementation* performed in the study by Bernardes et al. (2023) suggests that hibiscus increases the synthesis of butyrate, a metabolite that plays an important role in gut health and modulating inflammation. In addition, it reduced the presence of inflammatory cells and attenuated the formation of aberrant crypt foci, which are characteristic of cellular changes associated with the development of cancer. These findings indicate that hibiscus may have therapeutic potential in preventing preneoplastic lesions and fighting intestinal inflammation.

Data from the study conducted by El‐Shiekh et al. (2020) demonstrated that hibiscus has significant neuroprotective properties, being able to attenuate brain damage caused by the induction of Alzheimer's disease in mice. Hibiscus administration resulted in cognitive improvement, as evidenced by behavioral tests such as the object recognition test and the assessment of spatial memory. Supplementation was also associated with a reduction in brain inflammation and a decrease in the accumulation of amyloid plaques, characteristic of Alzheimer's disease.

The study on the bioavailability of bioactive compounds in the drink of *Hibiscus sabdariffa* L. as an anti‐inflammatory potential, conducted by Arce‐Reynoso et al. (2023), investigated the impact of the microbiota on the biotransformation of phenolic compounds present in the drink. The results indicated that the microbiota extensively modified the PCs, and their amount was lower than that of organic acids, particularly hibiscus acid and hydroxycitric acid. Colonic metabolites derived from PCs and organic acids would be responsible for the anti‐inflammatory bioactivity described for *Hibiscus sabdariffa* L.

Research on the anticancer effects of the compound found in *Hibiscus*, gossypetin, was carried out by Huang et al. (2023) demonstrated that this compound has anticancer activities, being able to inhibit the growth and proliferation of oral squamous cell carcinoma cancer cells. The administration of gossypetin led to a reduction in cell viability, promoting apoptosis in tumor cells.

However, in a study carried out by Rambe et al. (2022), the objective was to investigate the effect of rosella leaf extract gel ( *Hibiscus sabdariffa* L.) in wound healing. The results indicated that there were no significant differences in wound healing and epithelial thickness between the groups. Hibiscus leaf extract at a concentration of 15% showed higher healing properties based on clinical and histological evaluation. Although there were no statistically significant differences, the results suggest that *Hibiscus* leaf has potential to be studied for future wound healing therapies.

## 4 Discussion

The main constituents of *Hibiscus* in the context of therapeutic importance are a polysaccharide, organic acid, and flavonoids, mainly anthocyanins. Dried calyx extracts are known to contain chemical constituents such as organic acids (citric acid, ascorbic acid, maleic acid, hibiscus acid, oxalic acid, tartaric acid), as well as phytosterols, polyphenols, anthocyanins, and other water‐soluble antioxidants. Organic acids, together with bioactive components, have free radical scavenging activity. The beneficial effect on health is mainly attributed to these bioactive molecules (Riaz and Chopra 2018).

Recent clinical studies and reviews confirm that the consumption of *Hibiscus sabdariffa* can reduce inflammatory markers such as hs‐CRP, TNF‐α, and MCP‐1, in addition to improving metabolic and oxidative stress parameters, reinforcing its role as an anti‐inflammatory modulator in chronic conditions (Montalvo‐González et al. 2022). El Bayani et al. (2018) reported that HS extracts (at 500 mg/kg bw) exhibited potent anti‐inflammatory properties in excess rats and prevented spatial memory impairments, associated with the ability of bioactive compounds to maintain the ratio of IL‐1β/IL‐1ra levels in plasma and hippocampus.

The studies by Alsharif et al. (2021) and Zhang et al. (2015) demonstrate the therapeutic potential of protocatechuic acid (PCA) in lipopolysaccharide (LPS)‐induced acute lung injury (ALI). Alsharif et al. (2021) showed that pre‐administration of PCA significantly attenuates lung damage associated with the septic response, through its antioxidant, anti‐inflammatory, and anti‐apoptotic properties. In addition, Zhang et al. (2015) showed that the administration of PCA reduced pulmonary histopathological changes, the concentration of proteins in the bronchoalveolar fluid, and the overproduction of proinflammatory cytokines TNF‐α and IL‐1β. In addition, PCA at a dose of 30 mg/kg inhibited the activation of the p38MAPK and NF‐κB signaling pathways, central mechanisms in the modulation of inflammation. These results reinforce the role of PCA as a protective agent in experimental models of ALI, indicating its potential use in the treatment of inflammatory lung diseases.

In the study by Bernardes et al. (2022), it was tested that hibiscus ( *Hibiscus sabdariffa* L.) supplementation increases butyrate synthesis and reduces inflammatory cells. The authors reinforce the anti‐inflammatory effect of Hibiscus, suggesting that supplementation with dehydrated calyces of *H. sabdariffa* DHSC may be recommended to attenuate cellular responses in the early stage of preneoplastic lesions. Furthermore, in the field of oncology research, the study by Malacrida et al. (2022) showed that HsEF has antitumor effects in both breast tumor cells examined and that the involvement of ERα (Estrogen Receptor alpha) could explain the differences observed between the two cell lines. In another study, Huang et al. (2023) demonstrated that gossypetin present in *Hibiscus sabdariffa* L. has potential for use as a chemopreventive agent in oral cancer.

The research conducted by Mattioli et al. (2022) analyzed extracts of Olea *europea* L. leaves and petals of *Hibiscus sabdariffa* L., investigating the herbal mixture (HM) as the target of the application of intestinal motility dysfunction. It obtained the following results: HM can control ileum and colon contractility without blocking bolus progression. In addition, it has demonstrated the ability to selectively inhibit iNOS and has strong pro‐apoptotic activity against Caco‐2 cells.

It was verified in the study carried out by Sáyago‐Ayerdi et al. (2021) on the bioconversion of polyphenols and organic acids by the intestinal microbiota of predigested fructose of *Hibiscus sabdariffa* L., calyces and Agave ( *A. tequilana* Weber) evaluated in an in vitro dynamic model (TIM‐2) of the human colon, after HPLC‐ESI‐QToF‐MS analysis of samples collected at 0, 24, 48, and 72 h of fermentation, it was observed that hydroxycinnamic acids, flavonols, flavonols, and anthocyanins were mainly transformed into derivatives of hydroxyphenylpropionic, hydroxyphenylacetic, and hydroxybenzoic acids. In addition, organic acids, such as hydroxycitric and hibiscus acids, have been formed along with unidentified lactones and reduced compounds. Interestingly, no differences were observed between metabolites derived from microbes formed after Hb and Hb/AF fermentation. In conclusion, the polyphenol‐rich Hb colonic fermentation produces a wide range of microbial phenolic metabolites with potential health effects.

Analysis of the available studies reveals a paucity of research on the use of *Hibiscus sabdariffa* L. extract in diseases such as atherosclerosis. However, the study by Sun et al. (2022) that investigated the active compound of *Hibiscus sabdariffa* L. calyxes stands out: the delphinidin‐3‐o‐glucoside. The study was conducted in rabbits with atherosclerosis and aimed to evaluate whether DP (Delphinidin‐3‐O‐glucoside) could inhibit oxidative stress and inflammation in rabbits with atherosclerosis, two important factors in the progression of atherosclerosis. The results showed that DP alleviated oxidative stress and inflammation induced by HD in rabbits with atherosclerosis.

In the study by Herranz‐López et al. (2019), the LC‐HS extract significantly reduced lipid content and increased AMPK activity in a hypertrophied adipocyte cell model. Therefore, the consumption of 500 mg/day of polyphenol‐enriched LC‐HS extracts for 2 months in the context of an isocaloric diet by overweight individuals decreased symptoms associated with obesity‐related diseases. The modulation of fat metabolism in adipose tissue, probably mediated by AMPK activation, is proposed as a molecular target to be explored in future research.

These investigations have resulted in new discoveries, which enable the development of new strategies for use, dosage adjustments, and therapeutic approaches that suit the specific needs of each patient. These studies aim to deepen and expand the existing knowledge about the medicinal plant, elucidating its mechanisms of action, therapeutic potentials, and clinical applicability in a broader and more detailed way.

## 5 Conclusion

Based on a qualitative study of scientific productions on *Hibiscus sabdariffa* L. extract and its anti‐inflammatory properties, the analysis of the data revealed benefits in the use of the plant in several pathologies, including degenerative diseases, sepsis, neoplasms, intestinal dysfunctions, and atherosclerosis. Above all, the robust evidence of anti‐inflammatory properties, widely investigated in the therapeutic context, stands out. However, despite the promising results, studies on the application of *Hibiscus sabdariffa* L. in wound care and the healing process have not shown clear evidence of efficacy. This finding, based on the selected database, indicates a gap in scientific knowledge about the effects of Hibiscus in this specific aspect, highlighting the need for further investigations to evaluate its potential in wound healing.

## Author Contributions

**Anne Caroline Silva Nogueira da Cruz:** conceptualization (equal), formal analysis (equal), methodology (equal), writing – original draft (equal). **Michelline Joana Tenório Albuquerque Madruga Mesquita:** methodology (supporting). **Guilherme kauan Rocha Dantas:** visualization (equal). **Eduardo Rodrigues Silva:** methodology (supporting). **Victória Carvalho Falcone De Oliveira:** methodology (supporting). **Heitor Martins Rezende:** visualization (supporting). **Gustavo Medeiros Frota:** visualization (equal). **Mariana Cavalcante:** visualization (supporting). **José Renzo Castro Garcês:** methodology (supporting). **Marcelo Souza de Andrade:** supervision (supporting), validation (supporting), visualization (equal). **Maria do Socorro de Sousa Cartagenes:** formal analysis (lead), supervision (lead), validation (lead), visualization (lead).

## Conflicts of Interest

The authors declare no conflicts of interest.

## Data Availability

Data sharing does not apply to this article as no datasets were generated or analyzed during the current study.
